# Conceptualisation and Measurement of Social Cohesion within the Sport and Physical Activity Context: A Scoping Review

**DOI:** 10.3390/sports11120231

**Published:** 2023-11-22

**Authors:** Louis Moustakas, Jule Wagner

**Affiliations:** Institute for European Sport Development and Leisure Studies, German Sport University, 50933 Cologne, Germany

**Keywords:** sport for social cohesion, sport for development, social capital, survey, measurement

## Abstract

Sport, physical activity and social cohesion are increasingly linked within the academic literature. Indeed, studies recognise both the importance of social cohesion for promoting physical activity and the potential of sport to support social cohesion. Up until now, however, the ways in which social cohesion has been defined and measured in the context of sport and physical activity have not been the subject of much academic attention. Through a scoping review of studies measuring social cohesion in the sport and physical activity context, we aim to uncover how social cohesion is defined and measured, thus allowing us to better grasp how the concept is understood and operationalised in this field. As such, full-text inclusion occurred when studies quantitatively measured social cohesion through a questionnaire/survey instrument in connection with sport or physical activity participation or within programmes using sport to foster social cohesion. A total of 40 papers were included in the review, showing broad support for the argument that social cohesion is positively related to sport or physical activity participation. However, the retained texts engage on only a surface level with the concept of social cohesion, with around half not defining the term and the associated measurement tools using only a fraction of the dimensions typically associated with social cohesion. To conclude, we propose future directions to enhance conceptual engagement with and measurement of social cohesion.

## 1. Introduction

At its simplest, social cohesion is understood as the traits of a society that bind it together and allow it to move forward in a common direction. An often-debated and highly multi-dimensional concept, social cohesion is broadly understood as a combination of strong social relations, a sense of belonging, and an orientation towards the common good. Regardless of the exact conceptualisation, there is general agreement within the academic literature that social cohesion is an important component of promoting economic growth or peace [1], as well as addressing emerging crises such as the COVID-19 pandemic [2,3].

Relatedly, sport, physical activity and social cohesion are increasingly linked within this literature. Studies concerning the relationship with physical activity have become a significant strand within social cohesion research [4], and studies concerning the influence of sport activities or interventions on social cohesion have likewise increased in quantity sharply over the last 15 years [5]. This has come as policy, programmes and literature increasingly recognise the role of sport in potentially fostering social cohesion [6,7], as well as the importance of social cohesion in promoting sport and physical activity participation [8]. Indeed, on the one hand, sport is increasingly understood as having broad cross-cultural appeal and offering a dynamic, interactive setting that may help bring groups together and offer opportunities to support social cohesion [5]. On the other hand, social cohesion and its related sub-dimensions, such as strong social relationships, trust in others and a sense of belonging, are also understood as important facilitators of broader sport or physical activity participation [8].

Yet, although these connections are increasingly recognised, there are concerns about how rigorously the term is defined and conceptualised within sport-related literature. There are numerous competing definitions or conceptualisations of the term [9], but recent work has illustrated how the term can be ill-defined at both the programme level and within segments of the sport-related academic literature [5,10]. Similarly, in the broader field of health research, there are criticisms that researchers use the terms social capital and social cohesion interchangeably, although social capital can arguably be understood as only one dimension of the broader social cohesion picture [11,12,13]. Up until now, however, the ways social cohesion has been defined and, in particular, measured in the sport and physical activity contexts have not been the subject of much academic attention.

Definition and, by extension, measurement are essential to effectively validating the claims linking sport, physical activity, and social cohesion. Vague or absent definitions open up the risk that researchers or implementers enact their interpretations of social cohesion in measurement and evaluation without taking into account existing work on social cohesion, never mind local knowledge and perspectives [10,14]. Thorough definitions and associated measurements are also crucial to truly be able to isolate social cohesion and its sub-dimensions and understand the dynamic relationship between social cohesion, sport, and physical activity.

It is against this background that this paper is situated. Through a scoping review of studies measuring social cohesion in the sport and physical activity context, we aim to uncover how social cohesion is defined and measured, thus allowing us to better grasp how the concept is understood and operationalised in this field. In particular, we aim to document the definitions, dimensions and survey tools used in the studies. From this, we can better understand how social cohesion is conceptualised and measured, as well as further propose research and practical directions for this research area.

## 2. Methodology

Scoping reviews are a way of synthesizing knowledge in emerging or complex areas of study. In particular, scoping reviews can be used to map evidence associated with a topic, clarify concepts, and identify gaps [15]. As our research questions, which we expand on below, centre around understanding the definition and measurement of social cohesion in the sport and physical activity context, a scoping review was deemed appropriate.

For the following, we use the approach proposed by Arksey and O’Malley [16] to structure our review. As such, the review followed the five steps presented by the authors, namely: (1) identifying the research questions; (2) identifying relevant studies; (3) study selection; (4) charting the data; (5) collating, summarizing and reporting the results. Overall, our scoping review began in January 2023 and took approximately ten months to complete. In the following subsections, we detail the process associated with each of the five steps. Reporting of the results further aligns with PRISMA guidance on scoping reviews, and a PRISMA Scoping Review Extension checklist is provided within the supplementary materials.

### 2.1. Identification of the Research Questions

According to Arksey and O’Malley [16], research questions should guide the search strategy, not be overly narrow so that they limit the analytical process, and also be broad enough to identify relevant literature. As such, in line with the aims of our study, we developed three research questions to structure our work: (1) What tools are used to measure social cohesion in the sport and physical activity context? (2) What dimensions of social cohesion are reflected in these tools? (3) What is the relationship between sport, physical activity and social cohesion documented in the studies?

### 2.2. Determination of Relevant Studies

Numerous multidisciplinary and thematically relevant databases were selected, and various search combinations were piloted. A final search string was chosen ((“sport*” OR “physical activity” OR “leisure”) AND (“social cohesion”)) that balances the breadth and relevance of results as well as overall feasibility. Thus, terms often connected to social cohesion but that are distinct theoretical concepts, such as social capital or social inclusion, were not used to focus explicitly on the core concept at the heart of our research questions. Otherwise, the terms sport and physical activity are rather explicit and tend to capture literature in the physical education context. Finally, the term leisure was also adopted, as it helps to capture the literature concerning different forms of mobility undertaken for pleasure/health (e.g., walking or cycling for leisure) that can thus be broadly understood as physical activity.

Several high-quality databases, including Web of Science and SportDiscus, were used to generate results. Ultimately, databases were selected for their inclusion of relevant sport and health literature, as well as their ability to support data extraction for a review. Table 1 summarises the overall search terms and databases.

### 2.3. Study Selection

**Figure 1:**
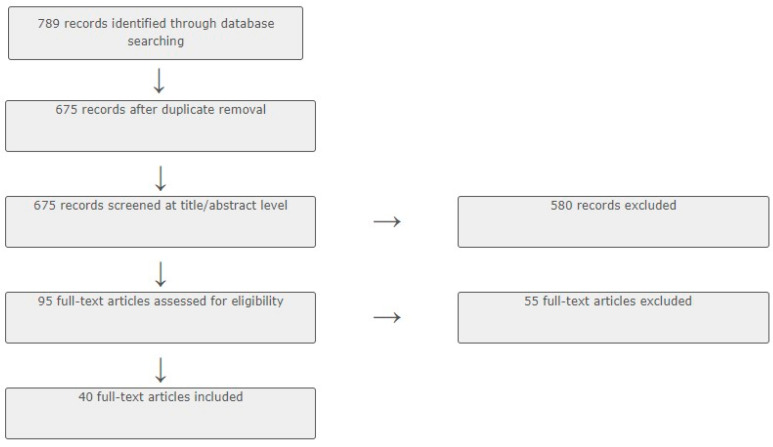
Prisma Flow Chart for Scoping Review.

Cadima software version 2.2.4 [17] was used to manage and streamline the process of abstract and full-text screening. Both authors reviewed each abstract and subsequent full-text, and a unanimous decision was required for texts to progress to full-text screening and, later, to full-text inclusion. Full-text inclusion occurred when studies quantitatively measured social cohesion through a questionnaire/survey instrument in connection with sport or physical activity participation, or within programmes using sport to foster social cohesion. Texts discussing social cohesion outside of the sport and physical activity context, such as in relation to transport, mobility, nutrition or sedentary behaviour, were excluded, as were texts relying on secondary data to assess social cohesion. In addition, using inbuilt functions within the respective databases, searches were limited to peer-reviewed journal publications, books, book chapters, and theses/dissertations published in English between 1994 and 2022. The full inclusion and exclusion criteria are summarised in Table 2, while the overall text selection process is depicted in Figure 1.

### 2.4. Charting the Data

The next stage of the process involved charting and data extraction from the included studies. We carried out this process using a shared spreadsheet and collected bibliographic and methodological information for all included studies. In terms of bibliographic and methodological information, we collected: author(s); year; journal; country of study; research questions; study design; and sample size and information. For the measurement instruments, we further looked at what tools were used to assess both physical activity as well as social cohesion. In particular, for the measurement of social cohesion, we charted the definition of social cohesion employed, the nature of the tool employed (e.g., self-designed or based on previous work), the social cohesion dimensions reflected in the tool, as well as any other social constructs captured in the study (e.g., neighbourhood aesthetics, self-efficacy, etc.)

To code the social cohesion dimensions embedded in the measurement tools, we used the dimensions of social cohesion from the conceptualisation put forth by the Bertelsmann Stiftung [18,19]. The work of this institution has served as the basis for many national and international studies (e.g., [20]) and breaks down social cohesion into three related dimensions. The first is social relations, which includes social networks, trust in people, and acceptance of diversity. The second is connectedness, which includes notions of identification, trust in institutions, and perception of fairness. Finally, there is a focus on the common good, which comprises ideas of solidarity, helpfulness, respect for social rules, and civic participation. These dimensions are outlined and described in Table 3. In addition, we coded for a dimension of shared values, as many definitions contend that shared values are important for social cohesion, though they do not always go into depth concerning the definition or nature of those values [9,21].

The first and second authors then undertook a pilot charting process that involved data extraction from three texts to become familiarized with the process and ensure consistency. Thereafter, charting was conducted by the second author, and both authors discussed any open questions and uncertainties to ensure continued consistency in the charting process. In addition, the first author randomly reviewed the charting of about 35% of the texts to verify quality and address emerging issues.

### 2.5. Collating and Reporting Results

Both frequency analysis and deductive coding were used to summarise and report the results. The variables extracted for the frequency analysis included: publication year, country of study, journal, study design, study sample, and measurement tools used. As discussed above, the deductive coding allowed us to identify the social cohesion dimensions in the measurement tools. Based on the results of this coding, we then conducted a frequency analysis to document the occurrence of each dimension.

## 3. Results

As depicted in the flow chart within Figure 1, from an initial pool of 789 records, 40 full texts were retained for extraction. As for the 55 excluded texts, the main reasons for exclusion included the use of secondary data (*n* = 32, e.g., national health surveys or census) and lack of investigation of the relationship between sport/physical activity and social cohesion (*n* = 11). Other reasons for exclusion included a lack of focus on social cohesion, a lack of focus on physical activity or sport, and non-English full texts. An overview of the retained texts, including bibliographic and methodological information, is presented in Table 4. Full results and analysis are described in the subsequent sub-sections.

### 3.1. General Study Characteristics

#### 3.1.1. Publication Year

Even though we set the timeframe for a review from 1994 onwards, no publications on the topic were found from before the year 2004. In total, the years of publication ranged from 2004 to 2022. Interest in the topic has risen over time, with 75% (*n* = 30) of the articles being published within the last decade (2012–2022).

#### 3.1.2. Journals

The articles were published in 24 different journals. *BMC Public Health* featured the most articles (*n* = 5), followed by *Preventive Medicine* (*n* = 4). Three articles each were published in the *Journal of Aging and Physical Activity* and *Social Science and Medicine*. Furthermore, two articles each were published in *Health and Place*, *Health Education and Behavior*, the *International Journal of Behavioral Nutrition and Physical Activity* and the *Journal of Physical Activity and Health*.

#### 3.1.3. Research Locations

The studies were conducted in 11 different countries on all 5 continents. Over half of the studies were conducted in the United States of America (*n* = 22). Consequently, with one additional study from Canada, North America was the continent where most studies took place (*n* = 23). Five studies each were conducted in Australia and Europe (France (1), Germany (1), Spain (1) and the Netherlands (2)). In Asia (China (2), Sri Lanka (1)) and South America (Brazil (3)), three studies were conducted. Only one study took place in Africa (Kenya). The research locations of the different studies indicate that research primarily focuses on the Global North, with only a few studies conducted in the Global South.

#### 3.1.4. Methodology

Most studies used a cross-sectional approach (*n* = 33). Two of the seven longitudinal study designs additionally employed a quasi-experimental setting with a control and an intervention group.

#### 3.1.5. Study Population

The sample sizes of the studies ranged from 39 to 14.924. A total of 12 samples contained less than 500 participants, and only 2 samples consisted of more than 10,000 participants. The average age of participants ranged from 3.8 years old to 74.5 years old. A total of 15 studies did not give a mean value for the age of its participants. The majority of studies were conducted with adults (*n* = 30) or both adolescents and adults (1). From these, seven studies focused on either middle-aged or middle-aged and older citizens (30–40-year-olds, up to 65–84-year-olds). Additionally, nine studies only looked at citizens aged fifty years and older. Consequently, nine studies were conducted with children, predominantly older children and adolescents (8–19-year-olds). Two studies also included smaller children aged two to seven.

The percentage of female participants ranged from 42.30% to 74%. Additionally, five studies specifically targeted women. Of these, two focused on mothers and two focused on Latina women living in the US. Furthermore, one study each investigated the experiences of Latino Americans, African Americans, and South Asian Americans, respectively.

### 3.2. Physical Activity

Only two studies did not use any measurement of physical activity. One of these examined the effect of an intercultural movement programme and thus did not have to measure physical activity separately [63]. The other study solely investigated which physical and social environment factors were associated with physical activity to develop a measurement tool [68].

A total of 23 studies used previously established tools to measure the physical activity of participants. The most commonly featured tool was the International Physical Activity Questionnaire (IPAQ), used in eight studies. A total of 15 studies used self-designed measurements to assess physical activity. The majority of studies relied on self- or caregiver-reported physical activity. However, five studies (additionally) used technical devices such as accelerometers to measure physical activity.

Most studies looked at physical activity in general, which was often divided into the categories of walking, moderate physical activity and vigorous physical activity. Seven studies solely examined the walking behaviour of participants and three studies only considered physical activity or play that happened outdoors. Furthermore, two studies included participation in recreational programmes or community-based activities in their physical activity measurement.

### 3.3. Definition and Measurement Tools for Social Cohesion

Over half of the articles did not give a clear definition of the term social cohesion (*n* = 24, 60%). Of the articles that defined social cohesion, seven used the definition of Kawachi and Berkmann [29], who define social cohesion as “the extent of connectedness and solidarity among groups in society”. Four articles followed Sampson, Raudenbush, and Earls’ [28] definition of social cohesion, which broadly views it as a combination of mutual trust and willingness to act for the common good. Furthermore, two articles used the definition of Chan, To, and Chan [45] who conceptualise social cohesion as “a set of attitudes and norms that includes trust, a sense of belonging and the willingness to participate and help” (p. 290). Four articles put forward their own definition of the concept.

To measure social cohesion, all but one study used statements about the neighbourhood to which participants had to state their level of agreement on an ordinal scale. A total of 75% of the studies employed or adapted already existing scales (*n* = 30). The most commonly used measurement tool, which was applied in 18 studies, was developed by Sampson, Raudenbush and Earls [28] and consists of five statements about the neighbourhood. It was followed by the social cohesion measure of Mujahid et al. [38], which features four statements and was used in five studies. Three studies used the items developed by Buckner et al. [35]. However, all of them employed a shorter version (5–8 items) of the original item list of 16 items. Two of the studies adapted the items themselves and one used the adapted version of the items of Buckner et al. [35] created by Seidman et al. [60]. Furthermore, two studies employed the social cohesion measurement tool of Friche et al. [23], consisting of six statements. The study of Pabayo, Janosz, Bisset and Kawachi used a 37-item tool previously developed by one of the authors and their colleagues to measure social cohesion at the school level [53]. The 6-item instrument used by Mendes de Leon et al. [48] was adapted from tools presented within three distinct papers. Furthermore, ten studies used self-designed measurements to examine social cohesion, with the number of items ranging from one to six. For two of the self-designed measures, only the number of items and an example were available, but not the complete list of items.

In summary, it seems to be common practice to use already existing measurement tools to assess social cohesion. Most papers used a relatively small number of statements (*n* < 9) to assess the social cohesion in the neighbourhood of participants. An overview of the measurement tools used and their social cohesion items (as far as available) can be found in Supplementary Table S1.

### 3.4. Dimensions of Social Cohesion

As most articles did not use a clear definition of social cohesion, the items of the measurement tools used in the studies are a good indicator for the understanding of social cohesion in the different articles. In general, the different statements about the neighbourhood used to assess social cohesion are often phrased similarly or go in the same direction.

**Figure 2:**
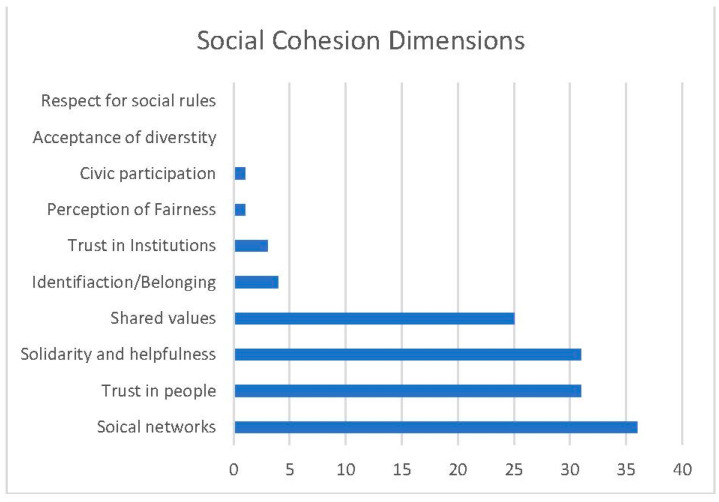
Dimensions of Social Cohesion captured by measurement tools within selected articles.

As shown in Figure 2, the most frequently captured dimensions of social cohesion were social networks (*n* = 36), trust in people (*n* = 31) and solidarity and helpfulness (*n* = 31), all of which were addressed by more than 75% of the studies. They were followed by the dimension of shared values, which was included in 25 studies (62.5%). In contrast, all the other dimensions of social cohesion were not or only rarely part of the measurement instruments. Identification/belonging was captured in four studies and trust in institutions in three studies. The dimensions of civic participation and perception of fairness were each included in one study. Acceptance of diversity and respect for social rules were not addressed in any of the measures explicitly related to social cohesion.

Relatedly, many studies measure other potentially similar social constructs, though not necessarily under the banner of social cohesion. For instance, three studies separately measured social capital, while other constructs such as neighbourhood aesthetics, collective efficacy, and availability of sporting services also appeared. Perceived safety, in particular, was prominent in studies investigating physical activity behaviour, appearing in 15 studies.

## 4. Limitations

Before progressing to our discussion, there are a few caveats and limitations that should be acknowledged. First and foremost, as this review is structured as a scoping review, quality assessment and meta-analysis of results did not occur. Thus, although we do make general statements about the outcomes within the included papers, we cannot speak to the overall quality of the evidence. Although omitting these steps was in line with the goals of our study and chosen methodology, we suspect future work could benefit from taking such steps. Second, we limited our search to conventional databases and English language literature. This means that we may have missed relevant research in other languages or emanating from more inclusive databases such as the Directory of Open Access Journals, Scielo, or African Journals Online. However, these aforementioned databases do not yet possess the same level of data extraction tools, thus making them less user-friendly in the context of a large review. Finally, our research terms focused specifically on social cohesion, but there is a well-known overlap between social cohesion, social capital and social inclusion, and additional usage of these terms may have uncovered other relevant measurement tools and approaches.

## 5. Discussion

This review illustrates how a large portion of the research does not define or engage in depth with the concept of social cohesion, and instead primarily relies on already existing survey measurement tools to conduct their studies. In turn, this reliance on a narrow set of pre-designed tools feeds into measurements that focus predominantly on dimensions related to social relations such as social networks and trust. Although this intense focus on only a handful of dimensions presents issues, which we will discuss later, the retained studies generally support the contention that social cohesion is a driver of physical activity, with 67.5% of the studies finding a significant positive relationship. As noted previously, quality assessment and meta-analysis of these results would help further confirm and expand our understanding of this relationship. Beyond that, however, from a research and more applied standpoint, our review points to numerous trends and implications within this context.

The most striking finding is the limited engagement with social cohesion as a concept. Most articles do not define the term, and the associated measurement tools connected to social cohesion focus predominantly on social relations. To be fair, other constructs were measured in these various articles, such as collective efficacy, trust, or social capital, and these constructs are typically understood as sub-dimensions of social cohesion as well. Yet, as a whole, this shows that authors investigating the connections between sport, physical activity and social cohesion mostly engage with the concept at a surface level, and do not take into account other facets of social cohesion that may have an important influence on sport or physical activity behaviour. Research shows that other factors, such as tolerance or trust in public institutions, play important roles in promoting physical activity. For instance, factors such as gender or migration background may limit sport participation [74], while, in contrast, the quality of public offers and facilities can be important promoters of physical activity [75]. This suggests that researchers would do well to fully engage with the concept of social cohesion and include questions related to issues of acceptance of diversity or trust in institutions, as these reflect important dimensions of social cohesion associated with sport and physical activity participation. Potential survey items already exist for these areas as well. Chan et al. [45] provide sample questions for these dimensions of social cohesion, and these could be further adapted for the sport and community contexts under study. However, until the full, multi-dimensional nature of social cohesion is fully accounted for, the ability of these studies to translate into meaningful evidence-based interventions or policy will remain limited [11], as the predominant focus on social relations risks minimising the crucial role of socio-political factors.

On the flip side, although there are a growing number of programmes attempting to use sport to support social cohesion, this review confirms the paucity of studies actively attempting to quantitatively measure programme outcomes. A separate review found that, of 35 papers, only 4 were using quantitative measurements [5], one of which is included here [63]. The other three, however, used social network analysis [76], employed behavioural measures [77], or merely represented a research protocol without any results [78]. In the adjacent field of sport for development (SFD), there are extensive debates about the suitability of various qualitative and quantitative methods, as well as a recognition of the need to integrate participants and stakeholders into research processes. Indeed, we fully agree that participants and communities should be made co-equal stakeholders in research and that such participatory approaches can ensure that research is reflective of and responsive to the realities of specific communities. However, conducting surveys or statistical analyses does not preclude these participatory or more critical approaches [79]. Rather, we would suggest that there is a need for extensive qualitative research in this field to be further complemented by more extensive quantitative work to provide combined, deeper insights. In that sense, we echo Massey and Whitley [80], who suggest that “rather than lay blanket critiques across different research paradigms and epistemologies, there is a need to discuss higher levels of sophistication in both instrumental/positivist (i.e., quantitative) and descriptive/critical (i.e., qualitative)” (p. 177) settings.

## 6. Conclusions

In this scoping review of 40 papers measuring social cohesion in the context of sport and physical activity, we found general support for the argument that social cohesion is positively related to sport or physical activity participation. Almost no quantitative measurements were located demonstrating a relationship between sport or physical activity participation or interventions and enhanced social cohesion. Furthermore, the retained texts engaged on only a surface level with the concept of social cohesion, with around half not defining the term and the associated measurement tools using only a fraction of the dimensions typically associated with social cohesion. For us, this highlights a need to holistically engage with the term and its measurement in order to generate deeper insights into the intersections between sport and physical activity.

## Figures and Tables

**Table 1:** Overview of search terms and databases.

Search Terms	(“sport” OR “physical activity” OR “leisure”) AND (“social cohesion”)
Search Area	Title, Abstract, Key Word
Databases	Web Of Science Web of Science Core Collection KCI-Korean Journal Database MEDLINE Russian Science Citation Index SciELO Citation Index EbscoHost SportDiscus Sociology Source Ultimate PSYINDEX

**Table 2:** Summary of inclusion/exclusion criteria.

	Inclusion	Exclusion
Topic	Texts quantitatively measuring social cohesion (i.e., via a questionnaire/survey) in connection with sport or physical activity participation or programmes. The relationship explored can be in either direction. Namely, studies can explore how social cohesion mediates sport/PA participation or look at how active participation in sport/PA influences social cohesion.	Texts measuring social cohesion outside of sport or physical activity contexts (e.g., mobility, nutrition, sedentary behaviour). Texts measuring similar but distinct concepts like social capital, team or group cohesion. Texts measuring social cohesion through secondary data (e.g., World Values Survey, Eurobarometer)
Population/Target Group	Target groups of all ages and backgrounds	None
Design/Form	Quantitative studies	Meta-analyses Systematic Reviews Conceptual or theoretical papers Position papers or editorials
Publication Type	Peer-reviewed journal articles Books Book chapters Theses/Dissertations	Grey Literature Proceedings Abstracts
Language	English	Documents not in English
Geographic Scope	Worldwide	None
Timeframe	1994–2022	Documents outside of the defined range

**Table 3:** Dimensions and sub-dimensions of social cohesion. Adapted with permission from [18].

Dimension	Sub Dimension	Description
Social relations	Social networks	Strong, resilient social networks.
	Trust in people	High level of trust in other individuals.
	Acceptance of diversity	Accept individuals with different backgrounds and lifestyles as equal members of society.
Connectedness	Identification	Individuals feel strongly connected with their geographic area and identify with it.
	Trust in institutions	Individuals have a high level of confidence in political institutions.
	Perception of fairness	Individuals believe that they are being treated fairly in society.
Focus on the common good	Solidarity and helpfulness	Individuals feel a responsibility for and willingness to help others.
	Respect for social rules	Individuals respect the fundamental rules of society.
	Civic participation	Individuals participate in society and civic and political life.

**Table 4:** Overview of included texts.

	**Bibliographic Information**			**Study Design and Methods**							
**Reference**	**Author(s)**	**Year (First Published)**	**Journal**	**Country of Study**	**Study Design**	**Sample Size**	**Average Age**	**Sample % Female**	**Measurement Instrument (PA)**	**Measurement Instrument (SoCo)**	**Definition Social Cohesion**
[22]	Rodrigues, D.E.; César, C.C.; Kawachi, I.; Xavier, C.C.; Caiaffa, W.T. and Proietti, F.A.	2018	Journal of Urban Health	Brazil	Cross-sectional survey	3667	41	59%	Leisure-time section of the long version of the International Physical Activity Questionnaire (IPAQ)	Based on Friche et al., 2012 [23]	No definition
[24]	Viana Peixoto, S.; Augusta de Lima Friche, A.; Lavalli Goston, J.; Comini César, C.; Coelho Xavier, C.; Augusto Proietti, F.; Diez Roux, A.V. and Teixeira Caiaffa, W.	2015	Cadernos de Saúde Pública/Reports in Public Health	Brazil	Cross-sectional survey	3597	41	53.10%	Leisure-time section of the long version of the International Physical Activity Questionnaire (IPAQ)	Based on Friche et al., 2012 [23]	No definition
[25]	Morata, T; López, P.; Marzo, T. and Palasí, E.	2021	International Social Work	Spain	Cross-sectional survey	203	n.a.	52.04%	Self-designed questionnaire: Leisure-based Community Activities and Social Cohesion (LCSC)	Self-designed questionnaire: Leisure-based Community Activities and Social Cohesion (LCSC)	Perception that the neighbourhood is a safe and cohesive environment. No citation provided
[26]	Liu, Z.; Kempermann, A. and Timmermans, H.	2020	Journal of Transport & Health	China	Cross-sectional survey	363	n.a.	52.30%	Self-designed questionnaire	Self-designed questionnaire	No definition
[27]	Ball, K.; Cleland, V.J.; Timperio, A.F.; Salmon, J.; Giiles-Corti, B. and Crawford, D.A.	2010	Social Science and Medicine	Australia	Cross-sectional survey	1405	n.a.	100%	Long version of the International Physical Activity Questionnaire (IPAQ) and self-reported walking	Based on Sampson et al., 1997 [28]	No definition
[8]	Cradock, A.L,; Kawachi, I.; Colditz, G.A.; Gotmaker, S.L and Buka, S.L.	2008	Social Science and Medicine	USA	Longitudinal interviews	680	n.a.	48.68%	Self-designed questionnaire	Based on Sampson et al., 1997 [28]	Kawachi and Berkman, 2000 [29]
[30]	Yuma-Guerrero, P.J.; Cubbin, C. and von Sternberg, K.	2017	Health Education & Behavior	USA	Cross-sectional survey	2750	n.a.	100%	Based on Kiernan et al., 2013 [31], only one question	Self-designed questionnaire	Kawachi and Berkman, 2000 [29]; Pebley and Sastry, 2004; Sampson et al., 2002 [32,33]
[34]	Rachele, J.N.; Ghani, F.; Loh, V.H.Y.; Brown, W.J. and Turrell, G.	2016	Preventive Medicine	Australia	Cross-sectional survey	10421 (5189 used as cases, 5232 as informants about area)	n.a.	55.20%	Based on Active Australia Survey	Based on Buckner et al., 1988 [35]	Social cohesion is a measure of social capital. No citation provided
[36]	Beenackers, M.A.; Kamphuis, C.B.M.; Mackenback, J.P.; Burdorf, A. and van Lenthe, F.J.	2013	Health Education Research	Netherlands	Cross-sectional survey	4395	n.a.	53.30%	Based on Dutch SQUASH questionnaire	Self-designed questionnaire	Kawachi and Berkman, 2000 [29]
[37]	Gao, J.; Hua, F.; Li, J. and Jia, Y.	2015	BMC Public Health	China	Cross-sectional survey	2783	n.a.	58.90%	Based on Chinese Version of the International Physical Activity Questionnaire (IPAQ)	Based on Mujahid et al., 2007 [38]	No definition
[39]	Van Dyck, D.; Teychenne, M.; McNaughton, S.A. and De Bourdeudhuij, I.	2015	PLoS ONE	Australia	Cross-sectional survey	3965	n.a.	52.30%	Based on long version of the International Physical Activity Questionnaire (IPAQ)	Based on Sampson et al., 1997 [28]	No definition
[40]	Joseph, R.P.; Vega-Lopez, S. and Han, S.	2021	JMIR Formative Research	USA	Cross-sectional survey	39	40.5	100%	Based on long version of the International Physical Activity Questionnaire (IPAQ)	Based on Mujahid et al., 2007 [38]	No definition
[41]	Yi, S.S.; Kanaya, A.M.; Wen, M.; Russo, R. and Kandula, N.	2021	Journal of Immigrant and Minority Health	USA	Cross-sectional survey	903	55.3	46.40%	Based on the Cross-Cultural Activity Participation Study	Based on Sampson et al., 1997 [28]	Neighbourhood social cohesion refers to the perceived network of relationships, shared values and norms of a neighbourhood. No citation provided
[42]	King, D.	2008	Journal of Aging and Physical Activity	USA	Cross-sectional survey	190	74.2	57%	Based on Community Health Activities Model Program for Seniors (CHAMPS)	Based on Sampson et al., 1997 [28]	Sampson et al., 1997 [28]
[43]	Samuel, L.J.; Dennison Himmelfarb, C.R.; Szklo, M.; Seeman, T.E.; Echeverria, S.E. and Diez Roux, A.V.	2014	Preventive Medicine	USA	Cross-sectional survey	5381	61.35	52.80%	Based on previous tool, which defined metabolic equivalent level of activity	Based on Sampson et al., 1997 [28]	Kawachi and Berkman, 2000 [29]
[44]	Cho, D.; Nguyen, N.T.; Strong, L.L.; Wu, I.; John, J.C.; Escoto, K.H.; Wetter, D.W. and McNeill, L.H.	2019	Health Education & Behavior	USA	Cross-sectional survey and health assessment	1467	45.19	74.64%	Based on the six-item International Physical Activity Questionnaire (IPAQ)	Self-designed questionnaire	Suglia et al., 2016 Chan, To and Chan, 2006 [45,46]
[47]	Fisher, K.J.; Li, F.; Michael, Y. and Cleveland, M.	2004	Journal of Aging and Physical Activity	USA	Cross-sectional survey and census data	582	73.99	68.60%	Self-designed questionnaire	Based on Sampson et al., 1997 [28]	Social cohesion is one of several other linked subconcepts generating social capital accumulation in a community. No citation provided
[48]	Mendes de Leon, C.F.; Cagney, K.A.; Bienias, J.L.; Barnes, L.L.; Skarupski, K.A.; Scherr, P.A. and Evans, D.A.	2009	Journal of Aging and Health	USA	Longitudinal survey	4317	74.5	61%	Based on the 1985 Health Interview Survey	Adapted from Balfour and Kaplan, 2002; Fisher et al., 2004; Sampson et al. 2002 [47,49]	Sampson et al., 1997 [28]
[50]	de Souza Moreira, B.; de Souza Andrade, A.C.; de Souza Braga, L.; de Carvalho Bastone A.; Lustosa Torres, J.; Furtado Lima-Costa, M.F. and Teixeira Caiaffa W.	2021	Journal of Aging and Physical Activity	Brazil	Cross-sectional survey	4027	n.a.	59.70%	Based on the six-item International Physical Activity Questionnaire (IPAQ)	Self-designed questionnaire	No definition
[51]	Jospeh, R.P. and Vega-Lopez, S.	2020	BMC Research Notes	USA	Cross-sectional survey	75	37.6	65.30%	Based on Rapid Physical Activity Questionnaire (RAPA)	Based on Mujahid et al., 2007 [38]	No definition
[52]	Pabayo, R.; Janosz, M.; Bisset, S. and Kawachi, I.	2014	PLoS ONE	Canada	Longitudinal survey	14924	n.a.	54.80%	Self-designed questionnaire	Based on Janosz et al., 1998 [53]	No definition
[54]	Baldwin, J.; Arundell, L. and Hnatiuk, J.A.	2022	BMC Public Health	Australia	Cross-sectional survey	214	3.8	42.30%	Based on the Australian Bureau of Statistics, Census of Population and Housing, 2016	Based on Sampson et al., 1997 [28]	No definition
[55]	Ganzar, L.A.; Salvo, D.; Burford, K.; Zhang, Y.; Kohl, H.W. and Hoelscher, D.M.	2022	International Journal of Behavioral Nutrition and Physical Activity	USA	Longitudinal survey	168	8.9	56%	Self-designed questionnaire	Based on Sampson et al., 1997 [28]	No definition
[56]	Yamamoto, M. and Jo, Hyerim	2018	Health & Place	USA	Cross-sectional survey	491	40	51.50%	Self-designed questionnaire	Based on Sampson et al., 1997 [28]	Sampson, 1991; Sampson et al., 1997 [28,57]
[58]	Rosenblatt, A.M.; Crews, D.C.; Powe, N.R.; Zonderman, A.B.; Evans, M.K. and Tuot, D.S.	2021	BMC Public Health	USA	Cross-sectional survey	2082	56.5	59.10%	Based on Baecke et al., 1982 physical activity questionnaire	Based on Sampson et al., 1997 [28]	No definition
[59]	Perez, L.G., Carlson, J.; Slymen, D.J.; Patrick, K.; Kerr, J.; Godbole, S.; Elder, J.P.; Ayala, G.X. and Arrredono, E.M.	2016	Preventive Medicine Reports	USA	Cross-sectional survey and anthropometric measurements	86	45.4	100%	Accelerometer and GPS device for 7 days	Based on Seidman et al., 1995 [60]	No definition
[61]	Dlugonski, D.; Das, B.M.; Martin, T. and Palmer, A.	2017	Family and Community Health	USA	Cross-sectional survey and anthropometric measurements	86	39.2	100%	Wearable activity monitor	Based on Sampson et al., 1997 [28]	No definition
[62]	Pabayo, R.; Belsky, J.; Gauvin, L.; Curtis, S.	2011	Social Science and Medicine	USA	Longitudinal survey	889	n.a.	49.9%	Accelerometer	Self-designed questionnaire	Kawachi and Berkman, 2000 [29]
[52]	Pabayo, R.; Molnar, B.E.; Cradock, A. and Kawachi, MD	2014	American Journal of Public Health	USA	Cross-sectional survey	1364	16.3	56.1%	Based on the Youth Behavior Surveillance System	Based on Sampson et al., 1997 [28]	Kawachi and Berkman, 2000 [29]
[63]	Grimminger-Seidensticker, E. and Möhwald, A.	2020	Physical Education and Sport Pedagogy	Germany	Longitudinal survey, quasi-experimental	227	10.8–11.6	43.36%	Not measured	Self-designed questionnaire	No definition
[64]	Silfee, V.J.; Rosal, M.C.; Sreedhara, M.; Lora, V. and Lemon, S.C.	2016	BMC Public Health	USA	Cross-sectional verbal assessment	602	46.64	51.2%	Based on Women’s Health Initiative (WHI) Brief Physical Activity Questionnaire	Based on Mujahid et al., 2007 [38]	No definition
[65]	Echeverria, S.; DIez-Roux, A.; Shea, S.; Borrell, L. and Jackson, S.	2008	Health & Place	USA	Cross-sectional survey	5943	61.8	52.0%	Self-designed questionnaire	Based on Sampson et al., 1997 [28]	Sampson et al., 1997 [28]
[66]	Karusisi, N.; Bean, K.; Oppert, J.-M.; Pannier, B. and Chaix, B.	2012	Preventive Medicine	France	Cross-sectional	7290	n.a.	n.a.	Self-designed questionnaire	Based on Mujahid et al., 2007 [38]	No definition
[67]	Aarts, M.-J.; Wendel-Vos, W.; van Oers, H.A.M.; van de Goor, I.A.M. and Schuit, A.J.	2010	American Journal of Preventive Medicine	The Netherlands	Cross-sectional survey	6470	7.8	49.88%	Self-designed questionnaire	Self-designed questionnaire	No definition
[68]	De Silva Weliange, S.H.; Fernando, D.; and Gunatilake, J.	2014	BMC Public Health	Sri Lanka	Cross-sectional survey	180	n.a.	57.20%	Not measured	Self-designed questionnaire	No definition
[69]	Michael, Y.L. and Carlson, N.E.	2009	International Journal of Behavioral Nutrition and Physical Activity	USA	Longitudinal survey, experimental	582	74	76.5% (intervention), 64.9% (control)	Based on Yale Physical Activity Scale (YPAS)	Based on Sampson et al., 1997 [28]	No definition
[70]	Ghani, F.; Rachele, J.N.; Loh, V.H..; Washington, S. and Turrell, G.	2019	International Journal of Environmental Research and Public Health	Australia	Cross-sectional survey	6643	n.a.	57.2%	Based on the Active Australia Survey	Based on Buckner, 1998 [35]	No definition
[71]	Li, F. and Fisher, K.J.	2004	Journal of Physical Activity and Health	USA	Cross-sectional personal interview survey	582	73.99	68.6%	Self-designed questionnaire	Based on Sampson et al., 1997 [28]	No definition
[72]	Muthuri, S.K.; Wachira, L.-J.; Onywera, V.O. and Tremblay, M.S.	2016	Journal of Physical Activity and Health	Kenya	Cross-sectional survey and anthropometric measurement	563	9–11.9	53.5%	Based on the United States Youth Risk Behaviour Surveillance System	Based on Sampson et al., 1997 [28]	No definition
[73]	Strong, L.L.; Reitzel, L.R.; Wetter, D.W. and McNeill, L.H.	2013	American Journal of Health Promotion	USA	Cross-sectional survey	1374	45.1	74.6%	Based on the six-item International Physical Activity Questionnaire (IPAQ)	Based on Sampson et al., 1997 [28]	Kawachi and Berkman, 2000 [29]

## Data Availability

Not applicable.

## Supplementary Materials

The following supporting information can be downloaded at: https://www.mdpi.com/article/10.3390/sports11120231/s1, Table S1: Overview of measurement tools and their social cohesion items.
