# Supplementary material for: Conceptualisation and Measurement of Social Cohesion within the Sport and Physical Activity Context: A Scoping Review
Source: Sports (Basel). 2023 Nov 22;11(12):231. doi: 10.3390/sports11120231 (PMC10747867; doi:10.3390/sports11120231)
Supplement: Supplementary file 1 [file sports-11-00231-s001.zip › sports-2705299-supplementary.pdf]

Supplementary Table S1. Overview of measurement tools and their social cohesion items.

**Authors (Year)**	**Measurement Items**
Sampson et al. (1997)	• People in this neighbourhood can be trusted<br>• This is a close-knit neighboorhoud<br>• People around here are willing to help their neighbours<br>• People in this neighbourhood generally get along with each other<br>• People in this neighbourhood share the same values
Mujahid et al. (2007)	• People around here are willing to help their neighbors<br>• People in my neighbourhood generally get along with each other<br>• People in my neighbourhood can be trusted<br>• People in my neighbourhood share the same values
Buckner et al. (1988)	• Overall, I am very attracted to living in this neighbourhood<br>• I feel like I belong to this neighbourhood<br>• I visit with my neighbors in their homes<br>• The friendships and associations I have with other people in my neighbourhood mean a lot to me<br>• Given the opportunity, I would like to move out of this neighbourhood<br>• If I needed advice about something I could go to someone in my neighbourhood<br>• I think I agree with most people in my neighbourhood about what is important in life<br>• I believe my neighbors would help me in an emergency

	• I feel loyal to the people in my neighbourhood<br>• I borrow things and exchange favors with my neighbors<br>• I would be willing to work together with others on something to improve my neighbourhood<br>• I like to think of myself as similar to the people who live in this neighbourhood<br>• I rarely have neighbors over to my house to visit<br>• A feeling of fellowship runs deep between me and other people in this neighbourhood<br>• I regularly stop and talk with people in my neighbourhood<br>• Living in this neighbourhood gives me a sense of community
Friche et al. (2012)	• Neighbors who would give advice if something bad happens<br>• Neighbors who would help financially<br>• Neighbors who would inform about an interesting opportunity of job<br>• People that you trust to leave the keys<br>• People that you trust to leave a family member in case of emergency<br>• People that you trust to lend things
Janosz et al. (1998)	• Relationship between students<br>• Relationship between students and teachers<br>• Education, security, Justice, Equity<br>• Staff members treat all students the same<br>• Membership and Belonging
Morata et al. (2003)	• Quality of life in the neighbourhood

	• Feeling of security<br>• Feeling that there are common projects and concerns in the neighbourhood<br>• Knowledge abut available sociocultural and leisure activities in the neighbourhood<br>• Rating of the leadership of the neighbourhood organisations for the common good
Liu et al. (2020)	• How many people in the neighbourhood do you know well enough to talk with<br>• How do you rate social relations with your neighbors
Yuma-Guerrereo et al. (2017)	• Respondents feel at home in their neighbourhood<br>• Their neighbors are willing to help one another<br>• Their neighbrs feel connected to each other<br>• Their neighbors get along<br>• Their neighbors share the same values<br>• Their neighbors can be trusted
De Souza Moreira (2021)	• Do you believe you can trust most people in your neighbourhood?
Pabayo et al. (2011)	• Which statement describes your neighbourhood? Most people keep to themselves; don't talk/visit to most people; talk/visit a lot with other people<br>• How many neighbors do you know well enough to visit<br>• How often do you get together with your neighbors
Aarts et al. (2010)	• People in the neighbourhood are willing to help each other<br>• The neighbourhood is a tight community

	• The people in the neighbourhood can be trusted<br>• In general, people in the neighbourhood get along well<br>• People in the neighbourhood share the same values<br>• There are many children living in the neighbourhood
De Silva et al. (2014)	• Good interaction between people in the neighbourhood<br>• Harmony between people in the neighborhood<br>• Respect each other<br>• Free of social disorder/disputes<br>• Helpful people in the neighbourhood<br>• Trustworthy people in the neighborhood
Mendes de Leon et al. (2009) adapted from Balfour et al., Fisher et al. and Sampson et al. (1997)	• Do you know them by name<br>• Neighbors with whom you can talk friendly<br>• Neighbors take care of each other<br>• Neighbors and friends talking outside<br>• 2 additional items
Cho et al. (2019)	• People in this neighbourhood can be trusted<br>• 4 additional items
Beenackers et al. (2013)	• People can be trusted<br>• Other additional items not available
